# Differential expression, function and response to inflammatory stimuli of 11β-hydroxysteroid dehydrogenase type 1 in human fibroblasts: a mechanism for tissue-specific regulation of inflammation

**DOI:** 10.1186/ar1993

**Published:** 2006-07-17

**Authors:** Rowan S Hardy, Andrew Filer, Mark S Cooper, Greg Parsonage, Karim Raza, Debbie L Hardie, Elizabeth H Rabbitt, Paul M Stewart, Christopher D Buckley, Martin Hewison

**Affiliations:** 1 Division of Medical Sciences, Institute of Biomedical Research, The University of Birmingham Medical School, Birmingham, UK; 2 Division of Immunity and Infection, Institute of Biomedical Research, The University of Birmingham Medical School, Birmingham, UK; 3 Division of Endocrinology, Diabetes and Metabolism, Cedars-Sinai Medical Center, Los Angeles, California, USA

## Abstract

Stromal cells such as fibroblasts play an important role in defining tissue-specific responses during the resolution of inflammation. We hypothesized that this involves tissue-specific regulation of glucocorticoids, mediated via differential regulation of the enzyme 11β-hydroxysteroid dehydrogenase type 1 (11β-HSD1). Expression, activity and function of 11β-HSD1 was assessed in matched fibroblasts derived from various tissues (synovium, bone marrow and skin) obtained from patients with rheumatoid arthritis or osteoarthritis. 11β-HSD1 was expressed in fibroblasts from all tissues but mRNA levels and enzyme activity were higher in synovial fibroblasts (2-fold and 13-fold higher mRNA levels in dermal and synovial fibroblasts, respectively, relative to bone marrow). Expression and activity of the enzyme increased in all fibroblasts following treatment with tumour necrosis factor-α or IL-1β (bone marrow: 8-fold and 37-fold, respectively, compared to vehicle; dermal fibroblasts: 4-fold and 14-fold; synovial fibroblasts: 7-fold and 31-fold; all *P *< 0.01 compared with vehicle). Treatment with IL-4 or interferon-γ was without effect, and there was no difference in 11β-HSD1 expression between fibroblasts (from any site) obtained from patients with rheumatoid arthritis or osteoarthritis. In the presence of 100 nmol/l cortisone, IL-6 production – a characteristic feature of synovial derived fibroblasts – was significantly reduced in synovial but not dermal or bone marrow fibroblasts. This was prevented by co-treatment with an 11β-HSD inhibitor, emphasizing the potential for autocrine activation of glucocorticoids in synovial fibroblasts. These data indicate that differences in fibroblast-derived glucocorticoid production (via the enzyme 11β-HSD1) between cells from distinct anatomical locations may play a key role in the predeliction of certain tissues to develop persistent inflammation.

## Introduction

The profound effects of glucocorticoids on the immune system have underpinned their widespread use as therapeutic agents for inflammatory diseases [1]. In addition to their therapeutic effects during pathological persistent inflammation, glucocorticoids are also known to play a key role in physiological responses directed at resolving inflammation at both systemic and tissue-specific levels [2,3]. These effects are mediated at a molecular level by the nuclear glucocorticoid receptor (GR) [4], but recent studies have indicated that GR signalling is rheostatically regulated through tissue-specific metabolism of GR ligands. Specifically, the interconversion of active and inactive glucocorticoids is catalyzed by the enzyme 11β-hydroxysteroid dehydrogenase type 1 (11β-HSD1), which is located on the luminal surface of the endoplasmic reticulum [5,6]. The bidirectional nature of 11β-HSD1 means that it has capacity for both reductase (that is, conversion of inactive cortisone to active cortisol) and dehydrogenase (that is, cortisol to cortisone) metabolism. However, in most physiological settings the enzyme exhibits predominant reductase activity as a consequence of the coincident expression of hexose-6-phosphate dehydrogenase (H6PDH), which facilitates enhanced, localized concentration of the cofactor for 11β-HSD1, namely NADPH (nicotinamide adenine dinucleotide phosphate, reduced form), within the endoplasmic reticulum lumen [7].

Expression of 11β-HSD1 occurs primarily in GR-rich tissues, where the enzyme acts to increase local levels of active glucocorticoids, thereby providing a system for autocrine induction of GR-mediated responses [5,6]. Prominent among these tissues is the liver, where glucocorticoids act as key metabolic regulators [8]. However, the enzyme is also abundantly expressed by cells such as adipocytes [9,10], osteoblasts [11], myocytes [12,13] and vascular cells [14], suggesting an additional role for 11β-HSD1 as a determinant of glucocorticoid responses in mesenchymal cells. This includes effects on cell proliferation [15], differentiation [9,10,16] and function [17], most notably in fat, where the enzyme appears to be a key facet of adipocyte differentiation [13,14] and insulin sensitivity [18]. The presence of 11β-HSD1 in fibroblasts was previously documented [19,20], particularly in the context of adipocyte differentiation [21]. Analysis of 11β-HSD1 in adipose stromal cells has revealed significant variations in enzyme expression between fat depots from different localities, suggesting that the enzyme is an important factor in site-specific responses to glucocorticoids [10,22]. However, the underlying impact of the enzyme in terms of mesenchymal stromal cell function has still to be fully defined. In studies presented here we examined site-specific expression of 11β-HSD1 in fibroblasts and show that the resulting variations in glucocorticoid metabolism play an important role in defining fibroblast cell phenotype and their functional responses to inflammation.

## Materials and methods

### Isolation and culture of human fibroblasts

All reagents used in cell culture were obtained from Sigma (Poole, UK) unless otherwise stated. Fibroblasts were isolated from biopsies of matched skin, bone marrow and synovium removed during total knee arthroplasty [23] from consenting patients who fulfilled the American College of Rheumatology (formally the American Rheumatism Association) criteria for rheumatoid arthritis (RA; *n *= 6) and osteoarthritis (OA; *n *= 3). Fibroblasts were isolated by mechanical digestion of tissue followed by dissociation in 5 mmol/l EDTA for 2 hours. Dissociated tissue was then washed and transferred to a culture flask. The fibroblasts were then cultured through to a maximum of seven passages in complete fibroblast medium consisting of RPMI-1640, 1% (vol/vol) non-essential amino acids, 1% penicillin/streptomycin, 1% sodium pyruvate, 2 mmol/l glutamine and 20% heat-inactivated foetal bovine serum (Labtech International, Sussex, UK) [23]. Fibroblasts were treated with 10 ng/ml IL-1, tumour necrosis factor (TNF)-α, IL-4 and interferon-γ (R&D Systems, Abingdon, UK) or 100 nmol/l cortisol or cortisone for 24 hours before harvesting.

### Immunohistochemistry

Cryostat tissue sections of synovial tissue from patients with RA were analyzed using goat polyclonal antibodies to 11β-HSD1 (The Binding Site, Birmingham, UK) with anti-sheep/goat biotin AB360 as secondary antibody (The Binding Site). Immunohistochemistry was also carried using polyclonal antisera to the following: the endothelial marker von Willebrand Factor (vWF; Dako, Ely, UK) with a secondary anti-rabbit AMCA antiserum (Jackson ImmunoResearch Laboratories, Inc., West Grove, PA, USA); the fibroblast marker ASO2 (CD90) with a secondary anti-mouse IgG_1 _Alexa 633 antiserum (Invitrogen, Paisley, UK); and the UCHT-1 (CD3) antiserum (gift from Peter Beverly, UCL, London, UK) with secondary anti-mouse IgG_2b _TRITC (Southern Biotech, Birmingham, USA). Immunohistochemical analyses and subsequent image visualization were carried out as described previously [24].

### RNA extraction and reverse transcription

RNA was extracted from cultured fibroblasts using the single-step extraction method (TRI Reagent, Sigma, Poole, UK). Aliquots (1 μg) of RNA were then reverse transcribed using random hexamers in a 20 μl volume, as stated in the manufacturer's protocol (Promega, Madison, WI, USA) [25].

### Real-time PCR

Expression of mRNA for 11β-HSD1, H6PDH, GRα, GRβ, IL-6, CCAAT/enhancer binding protein (C/EBP)α and C/EBPβ was assessed using real-time PCR in an ABI 7500 system (Applied Biosytems, Warrington, UK). The reactions were performed in 25 μl aliquots on a 96 well optical reaction plate (Sigma). Primers for 18S were used in conjunction with our target primers as an internal reference. Reactions contained TaqMan universal PCR master mix (Applied Biosytems), 900 nmol primers, 100–200 nmol TaqMan probe and 25–50 ng cDNA. Target genes probes were labelled with FAM and 18S probes were labelled with VIC. Reactions occurred as follows: 50°C for 2 minutes, 95°C for 10 minutes, 44 cycles of 95°C for 15 seconds and 60°C for 1 minutes. Data were obtained as Ct values (the cycle number at which logarithmic PCR plots cross a calculated threshold line) according to the manufacturer's guidelines (Applied Biosytems), and used to determine ΔCt values (Ct of target gene - Ct of housekeeping gene) as raw data for gene expression (high ΔCt = low gene expression). The fold change in gene expression was determined by subtracting ΔCt values for treated cells from their respective control samples. The resulting ΔΔCt values were then used to calculate fold change in gene expression according to the expression 2^ΔΔCt^. Probe and primer sequences used are summarized in Table 1.

### 11β-Hydroxysteroid dehydrogenase enzyme assays

Confluent cells in 12-well plates were cultured with 1 ml complete fibroblast medium, containing 100 nmol/l cortisone (to measure reductase activity) or cortisol (dehydrogenase activity) along with tritiated tracer for 18 hours. Steroids were extracted from growth medium using dichloromethane (5–10 vol) and separated by thin-layer chromatography (TLC) using ethanol:chloroform (8:92) as the mobile phase. TLC plates were analyzed using a Bioscan imaging detector (Bioscan, Washington, DC, USA) and the fractional conversion of steroids was calculated. In each case total protein concentration was assessed using a 96-well plate assay kit (Bio-Rad laboratories Inc., Hercules, CA, USA). Results were expressed as pmol product/hour per mg protein. All experiments were carried out in triplicate.

### Analysis of IL-6 and cortisol levels by ELISA

Cortisol levels in supernatants from cultured cells were measured using a commercially available sandwich ELISA in accordance with the manufacturer's instructions (R&D systems, Abingdon, UK). Data were expressed as pg cortisol/mg protein. Soluble IL-6 in supernatents from cultured cells was measured using a commercially available sandwich ELISA in accordance with the manufacturer's instructions (BD Biosciences Pharmingen, San Diego, CA, USA). Data were expressed as pg IL-6/mg protein.

### Statistical analysis

**Figure 1:**
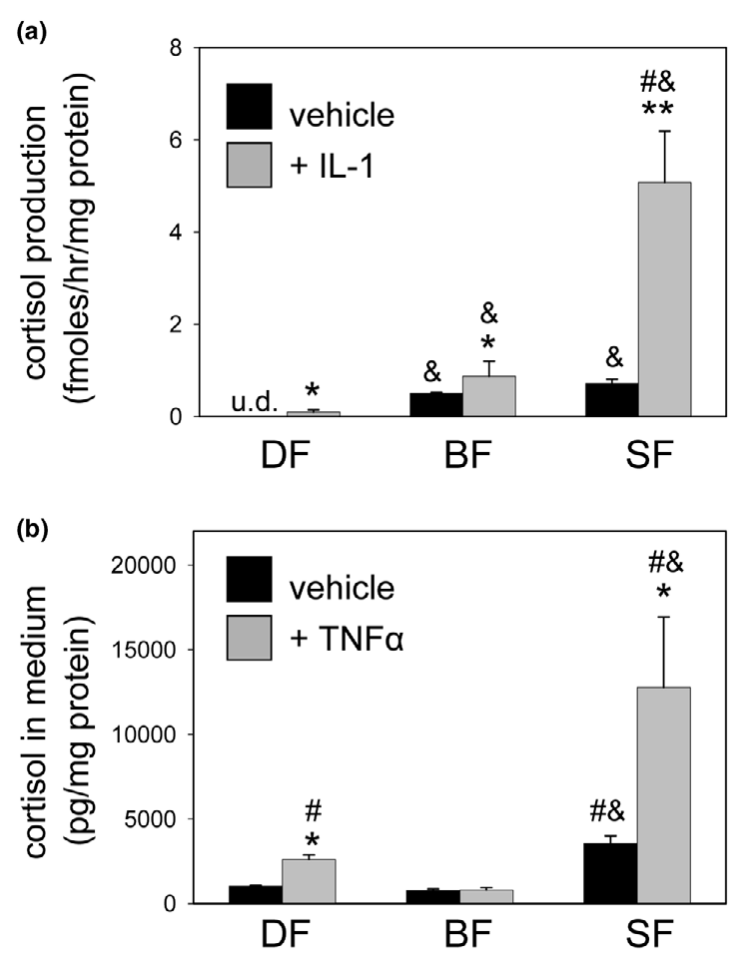
Tissue-specific expression of 11β-HSD1 is an autocrine determinant of cortisol levels in fibroblasts. Results are shown for cultures of dermal (DF), and bone marrow (BF) and synovial fibroblasts (SF). **(a) **Reductase activity determined by scanning thin layer chromatography (^3^H-cortisone to ^3^H-cortisol conversion, 12-hour incubation) following treatment with IL-1 (10 ng/ml, 24 hours) or vehicle. **(b) **Accumulation of cortisol in cell culture medium (as determined by specific ELISA) from 100 nmol/l cortisone following treatment with TNF-α (10 ng/ml, 24 hours) or vehicle. Values are expressed as mean ± standard deviation of four replicates from a representative culture of RA fibroblasts. Similar values were obtained when the assays were carried out using two other fibroblast cells lines. *P < 0.01, **P < 0.001 versus vehicle-treated cells; ^#^*P *< 0.01 versus equivalent bone marrow fibroblasts; ^&^*P *< 0.01 versus equivalent dermal fibroblasts. ELISA, enzyme-linked immunosorbent assay; IL, interleukin; TNF, tumour necrosis factor; u.d., undetectable.

**Figure 2:**
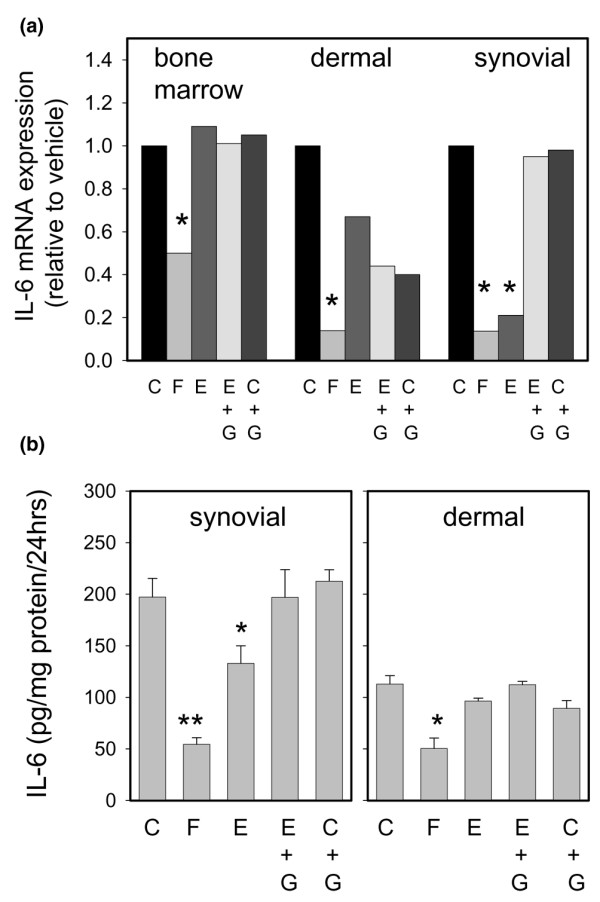
Autocrine activation of cortisol and the regulation of fibroblast function. Bone marrow, dermal and synovial fibroblasts were treated with vehicle (C), cortisol (F), or cortisone (E; both at 100 nmol/l) in the presence or absence of the 11b-HSD1 inhibitor glycyrrhetinic acid (+ G; 5 μmol/l) for 24 hours. Cells were then assessed expression of IL-6 mRNA and protein. **(a) **For mRNA analyses, target gene data were normalized for levels of the housekeeping gene 18S rRNA and presented as fold change in expression relative to vehicle-treated fibroblasts. **(b) **Analysis IL-6 protein secretion in synovial fibroblasts was carried out using a specific ELISA assay and reported as pg IL-6/mg cell protein/24 hours. Values are expressed as mean ± standard deviation of four replicates from a representative culture of rheumatoid arthritis fibroblasts. Similar values were obtained when the assays were carried out using two other fibroblast cells lines. *P < 0.01, **P < 0.001 versus vehicle-treated cells (statistical analysis carried out on unmodified ΔCt values). Ct = the cycle number at which logarithmic PCR plots cross a calculated threshold line; ELISA, enzyme-linked immunosorbent assay; 11β-HSD = 11β-hydroxysteroid dehydrogenase.

Data are reported as mean ± standard deviation (SD) of replicate mean values for separate patient cell cultures. However, because of interindividual variation, data in Figures 1 and 2 are shown as representative means (±SD) from single patient cell cultures. In these experiments assays were repeated at least twice using different cell preparations, with similar results. Regression analysis was performed using Microsoft Excel 2003. One-way analysis of variance was performed using SPSS Data Editor (SPSS Inc., Santa Clara, CA, USA).

**Figure 3:**
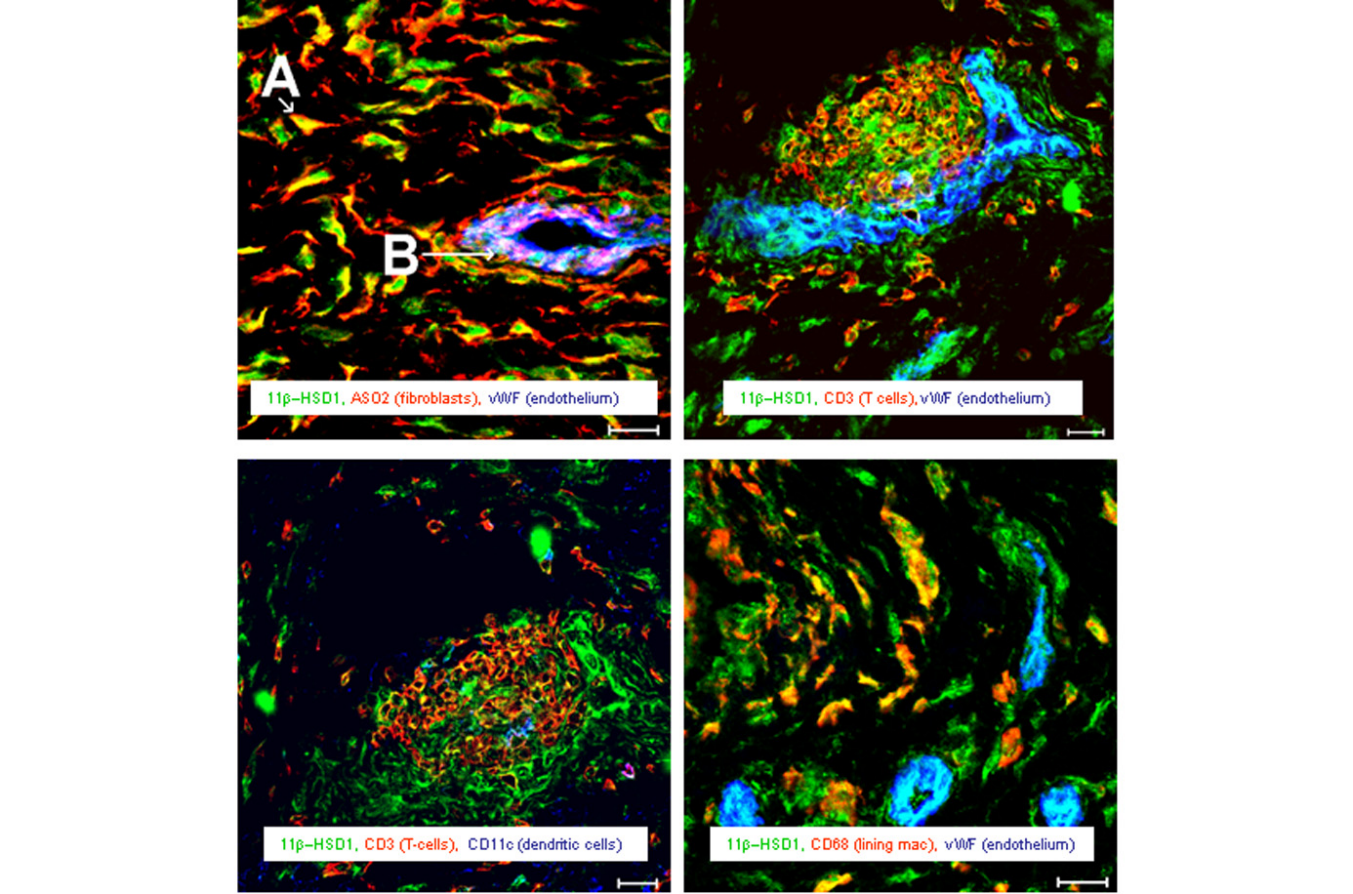
Expression of 11β-HSD1 in synovial tissue. Co-localization of 11β-HSD1 with markers of endothelial cells (vWF), fibroblasts (ASO2/CD90), T-cells (CD3), dendritic cells (CD11c) and lining macrophages (lining mac; CD68). Fluorescence immunohistochemistry was carried out using green, red and blue fluorescent tagged antiserum as shown in each panel. In each case the scale bar is 20 μm. A indicates co-expression of 11β-HSD1 and ASO2 (CD90) in fibroblasts; B highlights co-expression of 11β-HSD1 and vWF in endothelial cells. 11β-HSD, 11β-hydroxysteroid dehydrogenase type 1; vWF, von Willebrand factor.

We first examined whether synovial fibroblasts *in situ *expressed 11β-HSD1. Confocal immunohistochemistry (Figure 3) indicated that 11β-HSD1 was expressed by a variety of cells types in rheumatoid synovial tissue, including leukocytes such as T cells and macrophages. However, it was only weakly expressed by dendritic cells. The enzyme was abundantly expressed by CD90-positive fibroblasts and by endothelial cells (identified by von Willebrand factor staining).

**Figure 4:**
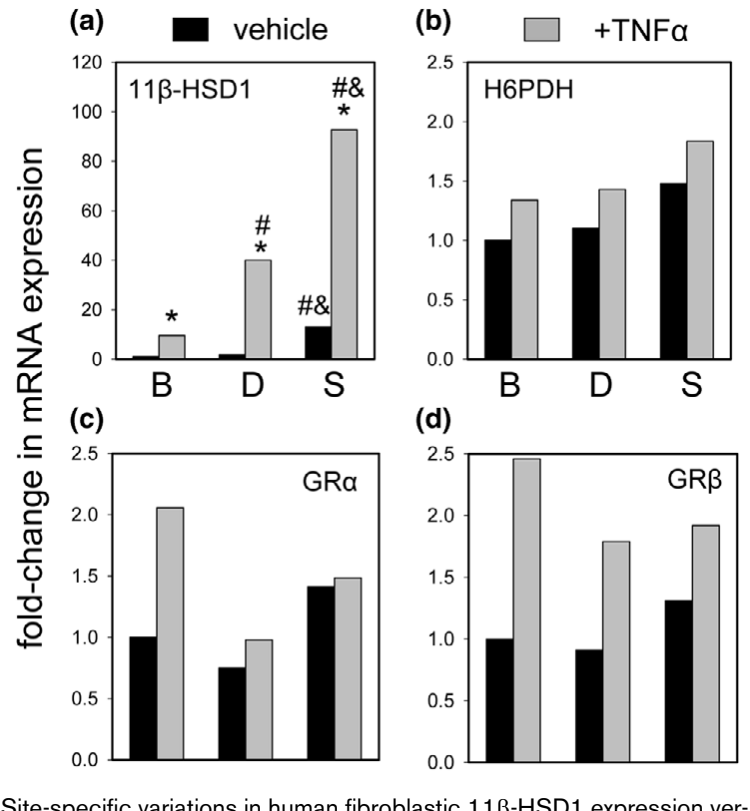
Site-specific variations in human fibroblastic 11β-HSD1 expression versus other components of glucocorticoid metabolism and signalling. Expression of mRNA for **(a) **11β-HSD1, **(b) **H6PDH, **(c) **GRα and **(d) **GRβ in bone marrow (B), and dermal (D) and synovial (S) fibroblasts in the presence or absence of TNF-α (10 ng/ml). For each gene product data were normalized for levels of the housekeeping gene 18S rRNA and are presented as fold change in expression relative to vehicle-treated bone marrow fibroblasts. *P < 0.01 versus vehicle control; ^#^*P *< 0.01 versus equivalent bone marrow fibroblasts; ^&^*P *< 0.01 versus equivalent dermal fibroblasts. 11β-HSD, 11β-hydroxysteroid dehydrogenase type 1; GR, glucocorticoid receptor; H6PDH, hexose-6-phosphate dehydrogenase; TNF, tumour necrosis factor.

To investigate the significance of 11β-HSD1 expression in fibroblasts, further studies were carried out using primary cultures of fibroblasts isolated from matched synovium, bone marrow and skin. Analysis of mRNA from these cells indicated that expression of 11β-HSD1 was greatest in synovial fibroblasts (ΔCt = 20.5 ± 2.2), followed by dermal (ΔCt = 23.3 ± 1.8) and bone marrow fibroblasts (ΔCt = 24.2 ± 0.9; Figure 4). In all three cell types expression of 11β-HSD1 was upregulated after treatment with TNF-α with the synovial fibroblasts (ΔCt = 17.7 ± 1.7) continuing to show the highest levels of mRNA relative to dermal (ΔCt = 18.9 ± 1.4) and bone marrow fibroblasts (ΔCt = 20.9 ± 1.2). These tissue-specific variations in baseline expression and potent inducibility by TNF-α were not observed for other components of glucocorticoid metabolism and signalling. For example, the NADPH-generating enzyme H6PDH and GRα exhibited similar levels of expression in the different fibroblast types, and this was not significantly affected by treatment with TNF-α (Figure 4). The nonfunctional GRβ was only weakly expressed in basal fibroblasts compared with GRα (ΔCt = 27.9 ± 2.1 in vehicle-treated versus 14.7 ± 1.9 for GRα) and was also unaffected by TNF-α treatment. Comparison of cells isolated from patients with RA (*n *= 6) with those from OA patients (*n *= 3) indicated that there was no significant difference in expression of 11β-HSD1, H6PDH, GRα, or GRβ mRNA in either vehicle or TNF-α-treated fibroblasts between the two disease types (data not shown).

**Figure 5:**
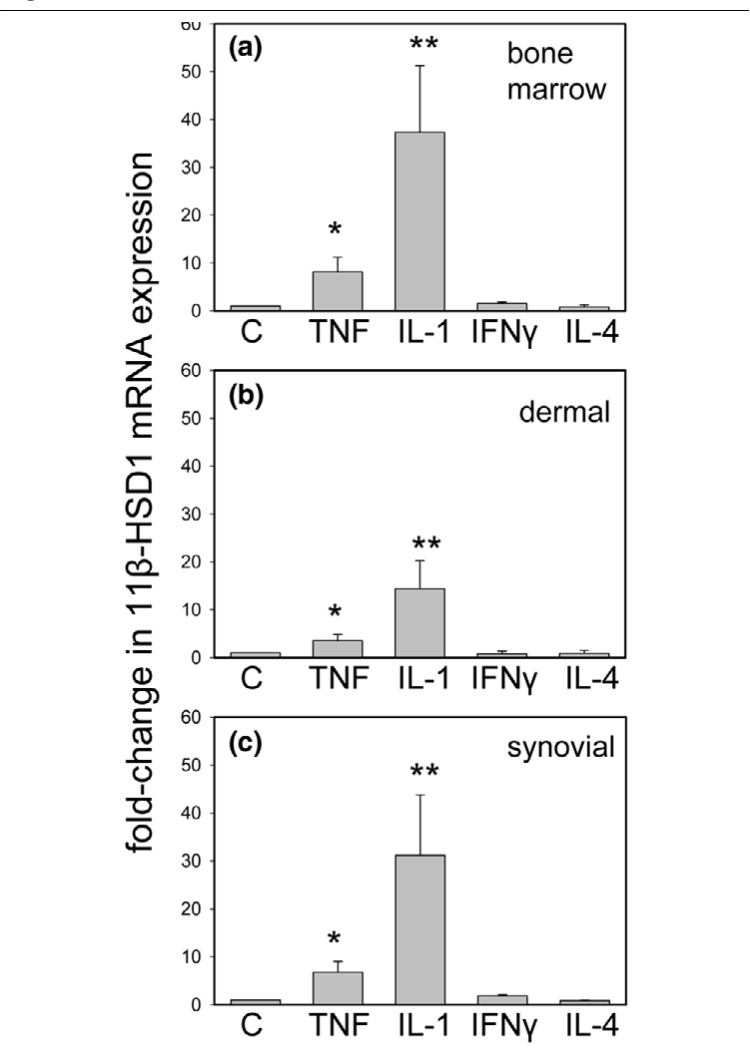
Induction of 11β-HSD1 expression is specific for inflammatory cytokines. **(a) **Bone marrow, and **(b) **dermal and **(c) **synovial fibroblasts (*n *= 3 for each) were cultured with or without TNF-α (10 ng/ml), IL-1 (10 ng/ml), IFN-γ (100 iU), or IL-4 (10 ng/ml) for 24 hours. Total RNA from these cells was then used to assess expression of mRNA for 11β-HSD1. In each case mRNA levels for 11β-HSD1 were normalized for the housekeeping gene 18S rRNA and presented as fold change (mean ± standard deviation) in expression relative to vehicle treated bone marrow, dermal, or synovial fibroblasts. *P < 0.05, **P < 0.01 versus vehicle-treated cells. 11β-HSD, 11β-hydroxysteroid dehydrogenase type 1; C, control; IFN, interferon; IL, interleukin; TNF, tumour necrosis factor.

Similar induction of 11β-HSD1 mRNA was seen with IL-1 in bone marrow (37-fold increase for IL-1 treatment and 8-fold increase for TNF-α treatment relative to vehicle), dermal (14-fold and 4-fold increase) and synovial (31-fold and 7-fold) fibroblasts (Figure 5). By contrast, classical T-helper-1 and T-helper-2 cytokines such as interferon-γ and IL-4 and had no significant effect on 11β-HSD1 expression in any of the fibroblasts.

The site-specific differences in fibroblastic 11β-HSD1 mRNA expression and differential induction by IL-1 and TNF-α were biologically relevant because they translated into differences in enzyme activity (Figure 1). Measurement of ^3^H-cortisone and ^3^H-cortisol metabolism by scanning analysis of TLC indicated predominant reductase activity in all three cell types, being highest in synovial fibroblasts, followed by bone marrow fibroblasts, and lowest in dermal fibroblasts (Figure 1a). Treatment with IL-1 (10 ng/ml, 24 hours) increased cortisol production in all three cell types at an order of magnitude that was similar to that seen for mRNA data (Figure 5). Further experiments were also carried out to confirm the predominant reductase activity (cortisone to cortisol conversion) of 11β-HSD1 in human fibroblasts. ELISA data to assess the absolute levels of cortisol in cell culture supernatants revealed a pattern of 11β-HSD1 activity similar to that determined by TLC analysis. Cortisol accumulation following 24 hours incubation with 100 nmol/l cortisone was again significantly higher in synovial fibroblasts and increased still further in cells that were pretreated with TNF-α (Figure 1b).

**Figure 6:**
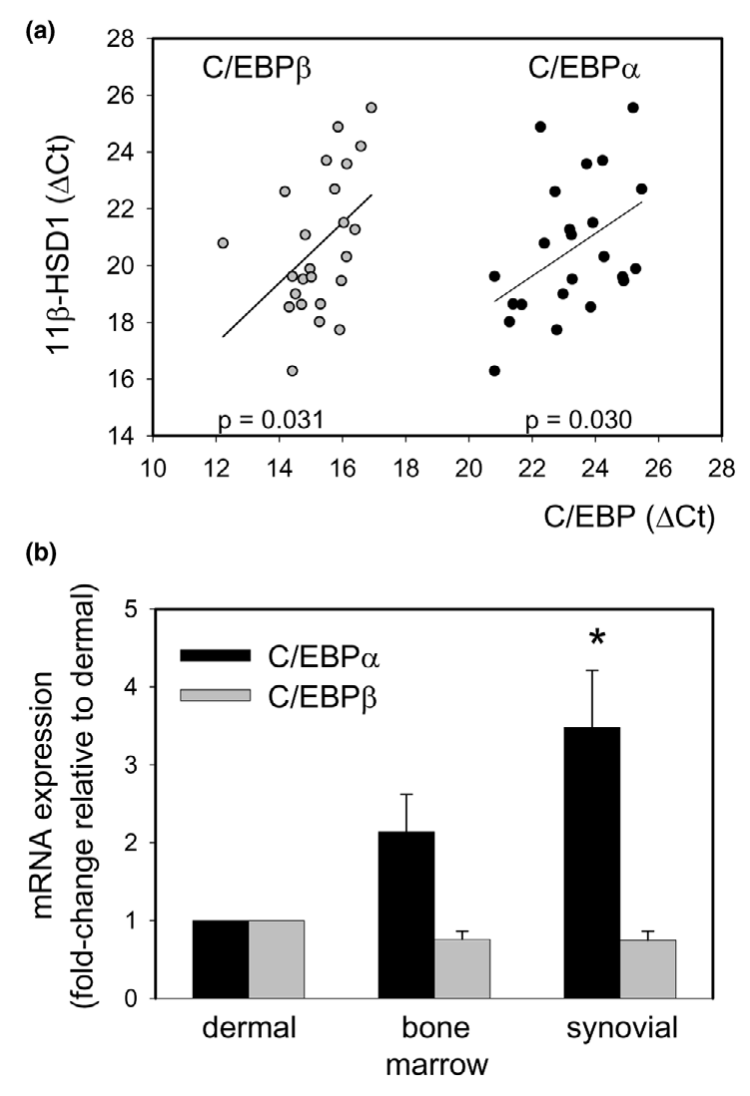
Expression of 11β-HSD1 in fibroblasts correlates with C/EBPα and C/EBPβ. **(a) **Correlation between ΔCt values for C/EBPα and C/EBPβ with ΔCt values for 11β-HSD1 in all fibroblast populations. **(b) **Fold change in C/EBP expression in bone marrow and synovial fibroblasts relative to dermal fibroblasts (mean ± standard deviation). *P < 0.01 versus dermal and bone marrow fibroblasts (statistical analysis carried out on ΔCt values). C/EBP = CCAAT/enhancer binding protein; Ct = the cycle number at which logarithmic PCR plots cross a calculated threshold line; 11β-HSD = 11β-hydroxysteroid dehydrogenase.

Previous studies have shown that members of the C/EBP family of transcription factors, namely C/EBPα and C/EBPβ, are potent regulators of 11β-HSD1 expression in hepatocytes [26]. A similar association between 11β-HSD1 and C/EBPα and C/EBPβ was also observed in fibroblasts (Figure 6a), although levels of C/EBPβ were significantly higher than the α isoform (higher ΔCt value = lower mRNA level). Both C/EBPα and C/EBPβ exhibited tissue-specific variations in expression (Figure 6b): C/EBPα was more strongly expressed in synovial fibroblasts, followed by bone marrow and then dermal fibroblasts, whereas the reverse pattern was observed for C/EBPβ.

The functional significance of tissue-specific variations in glucocorticoid metabolism was investigated by determining whether increased 11β-HSD1 activity was able to mediate autocrine regulation of the cytokine IL-6 in each of the different fibroblast types (Figure 2). Cortisol suppressed IL-6 mRNA levels in dermal (86%; *P *< 0.01), bone marrow (50%; *P *< 0.01) and synovial fibroblasts (87%; *P *< 0.01), indicating that all three cells had a similar capacity for GR-mediated responsiveness (Figure 2a). However, only synovial fibroblasts suppressed IL-6 mRNA expression when treated with the inactive glucocorticoid cortisone. This was completely reversed in the presence of the 11β-HSD inhibitor glycyrrhetinic acid, suggesting that cortisone achieves its effects on IL-6 via autocrine activation to cortisol (Figure 2a). Analysis of IL-6 protein secretion by ELISA in synovial fibroblasts confirmed that the findings observed at the RNA level were also reflected at the protein level. Because of variability in baseline IL-6 levels in different patients, data shown in Figure 2b were derived from a representative culture of RA fibroblasts. Similar results were observed in three sets of cells, and analysis of percentage change in IL-6 levels from these different cell preparations confirmed that in synovial fibroblasts both cortisol and cortisone were able to suppress IL-6 levels, with the effect of cortisone being abrogated by glycyrrhetinic acid (data not shown).

## Discussion

Fibroblasts and endothelial cells are able to influence immune responses through expression of specific cell adhesion molecules [27], cytokines [28] and chemokines [29] that collectively define the parameters required for entry, proliferation, survival and exit of leukocytes in a particular tissue. This stromal-derived 'address code' plays a key role in the initiation and resolution of inflammation but it is also an important factor in the disordered leukocyte trafficking associated with chronic persistent inflammation [30]. In previous reports we identified components of the stromal address code that are associated with specific tissues [31] and inflammatory responses [29]. Glucocorticoids are powerful endogenous modulators of inflammatory signal transduction [3], and because their effects vary between tissues we hypothesized that components of glucocorticoid action may also contribute to the stromal address code. Data presented here suggest that one of these factors, namely the glucocorticoid-metabolizing enzyme 11β-HSD1, is likely to play an important role in tissue-specific and inflammation-specific regulation of fibroblast function.

The predominant GR isoform, GRα, is ubiquitously expressed and mediates a wide range of responses to endogenous and exogenous glucocorticoids. Thus, dysregulated GR signalling is likely to be an integral component of persistent inflammation, and previous studies have associated glucocorticoid resistance with inflammatory disease [3,4]. In the present study we could detect no difference in GR expression between tissues or in response to cytokine treatment. Instead, striking variations in expression were observed for the enzyme 11β-HSD1, which exhibited cell-specific and cytokine-specific variations in expression and activity. Cytokine induction of stromal cell 11β-HSD1 has previously been reported in adipocytes [32], osteoblasts [33] and amnion-derived fibroblasts [20]. However, our data indicate that 11β-HSD1 expression and function are also subject to significant tissue-specific variations. Differential expression of 11β-HSD1 did not appear to be due to underlying inflammatory disease in donor RA patients, because similar observations were also made with cells from patients with OA. Furthermore, because fibroblasts from different sites were of matched donor origin, the effects of different exposure to drugs and disease duration can be excluded as confounding factors. It is possible that the method of fibroblast isolation could affect cell phenotype, but fibroblasts isolated in this way appear to maintain a pattern of gene expression to that seen *in vivo *[34].

There was a strong correlation between 11β-HSD1 and the transcription factors C/EBPα and C/EBPβ, which have been associated with hepatic expression of 11β-HSD1 [26]. C/EBPβ has been shown to be constitutively activated in RA synovial tissue but it does not appear to be involved in IL-1-induced expression of IL-6 or IL-8, both of which are more closely linked to induction of nuclear factor-κB [35]. We also observed no significant alteration in C/EBP expression in cells treated with proinflammatory cytokines, but our data suggest that tissue-specific variations in 11β-HSD1 may be dependent on the relative levels of C/EBP isoforms.

Although 11β-HSD1 is classically associated with hepatic glucocorticoid responses, its effects on stromal cells appear to be equally important as exemplified by the recent characterization of the adipocyte-specific transgenic mouse for 11β-HSD1, the phenotype of which is profound obesity [36]. In addition to this effect on mature cell function, the enzyme also appears to be intimately associated with stromal cell proliferation [15] and differentiation [16]. Differential expression of fibroblastic 11β-HSD1 may thus fulfil several functions. For example, the induction of the enzyme by IL-1 and TNF-α may be part of an autocrine feedback mechanism regulating inflammatory signalling. Autocrine metabolism of glucocorticoids has been identified in several cell types associated with immune responses, including macrophages [37], dendritic cells [38], synoviocytes [39], and lymphocytes [40]. Each of these different cell types appears to utilize 11β-HSD1 to attenuate distinct immune responses in an autocrine fashion [37,38,40], but the manner in which they are likely to interact in specific tissues remains unclear.

One recent study [39] suggested that synthesis of cortisol is reduced in cells from RA patients when compared with their OA counterparts, suggesting that a decreased availability of anti-inflammatory cortisol may contribute to the development or persistence of RA. This study reported expression and activity of 11β-HSD1 and suggested that this increased in response to inflammation, but the major difference between RA and OA tissues was the apparent expression of the glucocorticoid-inactivating enzyme 11β-HSD2 in nonfibroblastic cells. Although the nature and origin of the 11β-HSD2-expressing cells in RA is clearly of importance, it still appears that the normal response of synovial fibroblasts is to generate active glucocorticoids.

Exogenous glucocorticoids have a dramatic effect on synovial inflammation but their use is limited by systemic side effects. The data presented here indicate that there is an endogenous local counterpart that may play an important role in regulating synovial inflammation. It is possible that this system is needed to counteract endogenous inflammatory regulators (such as macrophage migration inhibitory factor [41]) that are more highly expressed in synovium than in other tissues. It is also possible that impairment of local glucocorticoid generation will adversely affect the inflammatory response within the joint. Although we saw no difference in the capacity of synovial fibroblasts from patients with RA or OA to generate active glucocorticoids, it is possible that variations in the timing of this response may contribute to the pathogenesis or severity of inflammatory arthritis. For example, local metabolism of prednisone/prednisolone may partly explain the early dramatic response to therapy that occurs as a result of increased generation of active glucocorticoids in affected tissues. Future studies of 11β-HSD1 *in vivo *will help to clarify its importance for the resolution, persistence and treatment of inflammation.

## Conclusion

Stromal cells play a pivotal role in normal, physiological inflammation and persistent inflammatory disease by expressing factors such as cytokines and chemokines that define local immune responses. We postulate that endogenous activation of glucocorticoids via the enzyme 11β-HSD1 is another important component of stromal cell function by providing an autocrine mechanism for the localized regulation of inflammation. Stromal cell 11β-HSD1 may also play a key role in responses to therapeutic glucocorticoids by increasing their concentration in a tissue-specific manner.

## Abbreviations

C/EBP = CCAAT/enhancer binding protein; Ct = the cycle number at which logarithmic PCR plots cross a calculated threshold line; ELISA = enzyme-linked immunosorbent assay; GR = glucocorticoid receptor; 11β-HSD = 11β-hydroxysteroid dehydrogenase; H6PDH = hexose-6-phosphate dehydrogenase; IL= interleukin; NADPH = nicotinamide adenine dinucleotide phosphate, reduced form; OA = osteoarthritis; PCR = polymerase chain reaction; RA = rheumatoid arthritis; SD = standard deviation; TLC = thin-layer chromatography; TNF = tumour necrosis factor.

## Competing interests

The authors declare that they have no competing interests.

## Authors' contributions

RSH cultured cells, extracted and analyzed RNA, and carried out enzyme activity studies. AF derived initial primary cultures. MSC developed initial hypothesis and co-wrote. GP derived initial primary cultures of fibroblasts. KR helped in the generation of cell lines and analyzed data. DLH carried out immunohistochemical analyses. EHR organized ELISA analyses. PMS developed initial hypothesis. CDB developed initial hypothesis, supervised collection of tissue and generation of cells, and co-wrote. MH developed initial hypothesis, supervised all cell analyses and co-wrote. All authors read and approved the final manuscript.

## Figures and Tables

**Table 1:** Probes and primer sequences used

Gene	Primers and probes
11β-HSD1	Forward primer: AGGAAAGCTCATGGGAGGACTAG
	Reverse primer: ATGGTGAATATCATCATGAAAAAGATTC
	Probe: CATGCTCATTCTCAACCACATCACCAACA
H6PDH	Forward primer: CAGGTGTCCTAGTGCACATTGAC
	Reverse primer: GTAGCCCACTCTCTCGTCCAA
	Probe: AAGGCACGCCCTCCCAGCG
GRα	Forward primer: GCGATGGTCTCAGAAACCAAAC
	Reverse primer: GAGATTACAGAGGAAGTTATCCTCTGC
	Probe: TGCAGTGAAGGTTGCTGAGGCTCTGA
GRβ	Forward primer: AAC TGG CAG CGG TTT TAT CAA CT
	Reverse primer: AACTCTTGGATTCTATGCATGAAAATGTTA TGTGGTTA
	Probe: TGT GTG AGA TGT GCT TTC TGG TT
C/EBPα	Forward primer: TGGACAAGAACAGCAACGAG
	Reverse primer: TTGTCACTGGTCAGCTCCAG
	Probe: CACCTTCTGCTGCGTCTCCACGTT
C/EBPβ	Forward primer: GACAAGCACAGCGACGAGTA
	Reverse primer: GTGCTGCGTCTCCAGGTT
	Probe: ATCTTGGCCTTGTCGCGGCTCTT
IL-6	Assay on demand Hs00174131_m1 (ABI)

11β-HSD1, 11β-hydroxysteroid dehydrogenase type 1; C/EBP, CCAAT/enhancer binding protein; GR, glucocorticoid receptor; H6PDH, hexose-6-phosphate dehydrogenase.
